# Vitamin D Deficiency in Children and Adolescents

**DOI:** 10.4274/jcrpe.574

**Published:** 2012-03-08

**Authors:** Nesibe Andıran, Nurullah Çelik, Halise Akça, Güzide Doğan

**Affiliations:** 1 Fatih University Faculty of Medicine, Department of Pediatric Endocrinology, Ankara, Turkey; 2 Fatih University Faculty of Medicine, Department of Pediatrics, Ankara, Turkey; +90 506 587 14 82haliseakca@gmail.com

**Keywords:** Vitamin D, deficiency, frequency, ferritin, risk factors

## Abstract

**Objective** Vitamin D deficiency is an important health problem in both developed and developing countries. Recent reports on the extraskeletal effects of vitamin D have led to increased interest in prevalence studies on states of deficiency/insufficiency of vitamin D. The aim of this study was to determine the frequency of vitamin D deficiency and insufficiency in children and adolescents residing in Ankara, Turkey and to investigate the factors associated with low vitamin D status.

**Methods:** A total of 440 children and adolescents aged between 0 and 16 years were enrolled in this study. The subjects were divided into three groups according to their vitamin D status (deficiency ≤15 ng/mL; insufficiency: 15-20 ng/mL; sufficiency ≥20 ng/mL) and also according to their age (preschool<5 years; middle childhood: 5-10 years; adolescence: 11-16 years).

**Results:** Overall, 40% of the subjects were found to have 25 hydroxy vitamin D [25(OH)D] levels of less than 20 ng/mL. The levels indicated a deficiency state in 110 subjects (25%) and insufficiency - in 66 (15%). The rate of vitamin D deficiency was higher in girls, regardless of age. 25(OH)D levels correlated negatively with age (r=-0.48, p<0.001), body mass index (BMI) (r=-0.20, p=0.001) and intact parathyroid hormone (iPTH) level (r=-0.31, p=0.001). A positive correlation was observed between 25(OH)D and serum ferritin levels (r=0.15, p=0.004).

**Conclusions:** The high frequency of vitamin D deficiency in childhood (especially among adolescent girls) indicates a need for supplementation and nutritional support.

**Conflict of interest:**None declared.

## INTRODUCTION

Vitamin D deficiency is an important public health problem in both developed and developing countries, with a reported worldwide prevalence of 30-80% in children and adults (1,2,3). The role of vitamin D in bone mineralization is well-documented. However, only recently, numerous studies have reported a link between vitamin D deficiency and several chronic disorders such as type 1 diabetes mellitus (T1DM), systemic lupus erythematosus (SLE), multiple sclerosis (MS), cardiovascular disease (CVD) and several malignancies (4,5,6,7,8). These recent findings have led to greater emphasis on treatment of vitamin D deficiency and/or vitamin D supplementation.

Prevalence studies on vitamin D deficiency and/or insufficiency in the Turkish population, especially among prepubertal children over 3 years of age, are relatively scarce (9). The aim of this study was to determine the frequency of deficiency and insufficiency of vitamin D in a sample of Turkish children and adolescents and to investigate the factors associated with low vitamin D status. 

## METHODS

This study was undertaken by the Fatih University Pediatrics Department and conducted in the University hospital, situated in Ankara, Turkey. We retrospectively reviewed the records of a total of 440 children and adolescents aged between 0 and 16 years who had presented to the outpatient clinic between January 2008 and January 2010. All patients were subjected to a careful physical examination. Weights were measured using a calibrated digital scale. Height measurements were done in triplicate to the nearest millimeter using a calibrated stadiometer. Body mass index (BMI) were calculated according to the formula [weight (kg)/height (m)2]. Obesity was defined as the presence of a BMI above the 95th percentile for age and sex based on established percentile curves for Turkish children (10). Patients with a history of a chronic disorder or on any medication that may alter vitamin D metabolism were excluded from the study. 

Venous blood samples were obtained for all patients from the antecubital region between 8.00-8.30 am after an 8-12-hour overnight fast. Serum calcium (Ca), phosphorus (P), magnesium (Mg), alkaline phosphatase (ALP) and glucose levels were measured using the enzymatic colorimetric method (Roche Integra 800), while serum 25 hydroxy vitamin D [25(OH)D] levels were measured by high-performance liquid chromatography (Shimadzu UFLC). Serum levels of ferritin, insulin and intact parathyroid hormone (iPTH) were evaluated using the electrochemiluminescence method (Advia Centaur XP). Insulin resistance for each patient was estimated by the homeostatic model assessment method insulin ratio (HOMA-IR) using the formula [HOMA-IR=(glucose X insulin)/405]. Vitamin D levels were estimated immediately after taking the blood sample. The same assays were used throughout the study. 

For evaluation of the results, the subjects were divided into three groups according to their vitamin D status (deficiency ≤15 ng/mL; insufficiency: 15-20 ng/mL; sufficiency ≥20 ng/mL) (11) and also according to their age (preschool <5 years; middle childhood: 5-10 years; adolescence: 11-16 years).

## STATISTICAL ANALYSIS

Statistical analysis was performed using SPSS 13.0 for Windows (SPSS, Inc., Chicago, IL, USA). One-way ANOVA was used in comparing variables with normal distribution. Pearson’s chi-square and Fisher’s exact tests were used for comparisons of categorical variables. Correlations between two variables were evaluated by Pearson’s correlation coefficient. Values for all parameters, except gender, were expressed as mean±standard deviation, and a p-value of less than 0.05 was considered indicative of statistical significance.

## RESULTS

**Figure 1:**
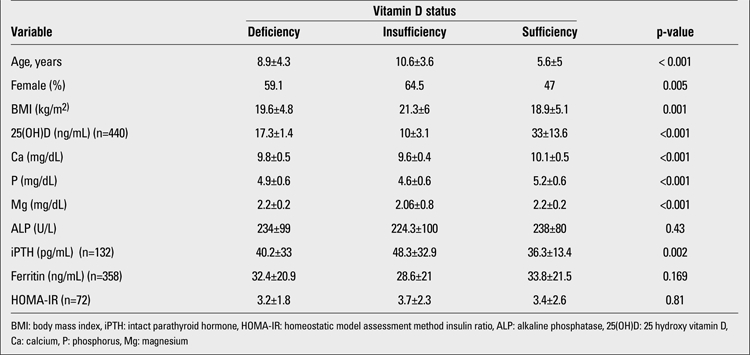
25(OH)D levels in the three age groups

**Figure 2:**
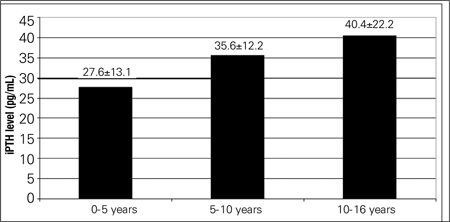
Intact parathyroid hormone levels in the three age groups

We retrospectively reviewed the records of a total of 440 children and adolescents aged between 0 and 16 years of age. Overall, 40% of patients had 25(OH)D levels lower than 20 ng/mL with 110 patients (25%) having deficiency and 66 patients (15%) having insufficiency. Comparisons between age groups in terms of vitamin D levels revealed that infants had a higher mean level of 25(OH)D compared to older children, with fewer of them having insufficiency (0-5 age group, 34.2±16.2 ng/mL; 5-10 age group, 20.5±8.7 ng/mL;10-16 age group, 18.7±11.5 ng/mL; Figure 1). Comparisons between age groups in terms of iPTH levels are shown in Figure 2.While 64.8% of adolescent girls had serum vitamin D levels below 20 ng/mL, this finding was observed in only 52.1% of adolescent boys. The rate of vitamin D deficiency was higher in girls, regardless of age group. The anthropometric and metabolic characteristics of the study population are given in Table 1.

Children with vitamin D deficiency were significantly older than those with normal serum 25(OH)D levels (10.3±3.6 years vs. 5.6±5 years; p=0.001). On the other hand, children with vitamin D deficiency had significantly higher BMI compared to patients with higher serum levels of 25(OH)D (Table 1). Correlation analyses revealed that 25(OH)D levels correlated negatively with age (r=-0.48, p<0.001), BMI (r=-0.20, p=0.001), and iPTH (r=-0.31, p=0.001), whereas a positive correlation was observed with serum ferritin levels (r=0.15, p=0.004). No correlation was found between serum vitamin D levels and HOMA-IR (r=-0.18, p=0.12). Results of correlation analyses are presented in Table 2. Lower serum Ca and P levels, but higher levels of ALP were observed in patients with vitamin D deficiency (<15 ng/mL). Significantly higher levels of iPTH were also observed in this group, indicative of the presence of a compensatory response to low 25(OH)D levels (Table 1).

## DISCUSSION

We found a remarkably high prevalence of poor vitamin D status among the 0-16 years age group included in this sample. This finding clearly indicates that vitamin D deficiency is an important health problem in children living in Ankara. However, one weak point of our study was that the prevalence was not determined on a population basis. We believe that a nationwide study would give more accurate results on the true prevalence. 

Vitamin D deficiency was found to be higher in girls than in boys, especially in the adolescent age group. It was also noted that the frequency of vitamin D deficiency increased with age. 

Studies on adults and children have shown that vitamin D insufficiency is more prevalent among overweight and obese people (12,13,14,15). Similarly, vitamin D deficiency was found to be more frequent in the obese subjects of our study. The etiopathogenesis of vitamin D deficiency in obese people is not clear. The sequestration of vitamin D in the subcutaneous body fat and its consequent reduced bioavailability was suggested to explain this relationship (16). Fewer outdoor activities and reduced sunlight exposure in obese individuals have also been suggested to contribute to reduced endogenous vitamin D production (17). In adults, low vitamin D status has been shown to contribute to development of cardiovascular diseases (18). A recent comprehensive study has shown that excess body weight at 14-19 years may increase cause-specific mortality in middle age (19). Therefore, the low vitamin D status observed in adolescents with excess body weight may be a contributing factor to this cause-specific mortality effect (15). 

We observed lower deficiency rates in the younger age group in our sample. This finding is probably related to preventive measures in primary health care conducted by the Ministry of Health in Turkey. Since 2005, vitamin D supplements are distributed to every newborn throughout their infancy at no financial cost (20). Furthermore, families and physicians tend to use multivitamins more frequently in younger age groups. In a study conducted in Israel, frequency of vitamin D deficiency was also found to be lower in the age group between 0 and 5 years compared to other age groups (3). Similar findings have also been recently reported by large demographic analyses conducted in the U.S. (21).

It has been reported that vitamin D insufficiency is more prevalent among girls (3,21). In a group of Turkish girls aged 14-18 years, prevalence of vitamin D insufficiency was reported to vary between 15.6% and 59.4%, according to socio-economic status and season (22). In our sample, considering all age groups, we found vitamin D deficiency to be higher in the girls. Unlike the study by Oren et al (3), vitamin D deficiency in girls was recorded also in the older age groups in our study. Vitamin D levels were below 20 ng/mL in 64.8% of adolescent girls between 10 and 16 years of age. This may be due to the clothing habits in Turkish girls and/or less time spent on outdoor activities (23). Recent studies suggest that vitamin D deficiency is particularly common among young women who wear concealing clothing (23,24) and that these young women are also at increased risk for osteoporosis (25,26).

The limited number of studies conducted on this issue have shown a positive correlation between the levels of 25(OH)D and ferritin. Constantini et al (27) reported a positive correlation between the levels of 25(OH)D and ferritin in athletes and dancers. Similarly, a positive correlation was reported between these two parameters in patients with Crohn’s disease (28). In our study also, ferritin level was likely to be lower in individuals with low 25(OH)D levels. This relationship may be explained by suboptimal dietary habits (27). This link is further supported by studies showing that 1α, 25-dihydroxycholecalciferol, an active form of vitamin D, leads to an increase in intestinal Fe absorption by elevating the erythropoietin level (29). The above-mentioned studies indicate that periodic screening should be performed for both vitamin D deficiency and low ferritin levels, two insidious conditions of high prevalence. 

In conclusion, vitamin D deficiency and insufficiency were found to be highly prevalent in our study group of children aged 0-16 years. This finding is in line with the high rate of vitamin D insufficiency recorded in Turkish infants and children, especially in girls and adolescents reported recently. The insufficiency state may be caused by low sunshine exposure, skin pigmentation, air pollution, skin covering and low vitamin D intake. According to our study, vitamin D supplementation should be provided not only for infants, but also for children of all ages, including adolescents.

## CONCLUSION

We concluded that vitamin D deficiency and insufficiency are highly prevalent in children aged 0 to 16. In our country, a high rate of vitamin D insufficiency is recorded in all age groups of children, especially in girls and adolescents. It may be caused by low sunshine exposure, skin pigmentation, air pollution, skin covering and low vitamin D intake. According to our study, it seems that vitamin D supplement should be provided not only for infants, but also for all the children, especially for those of in adolescent period. 

## Figures and Tables

**Table 1:**
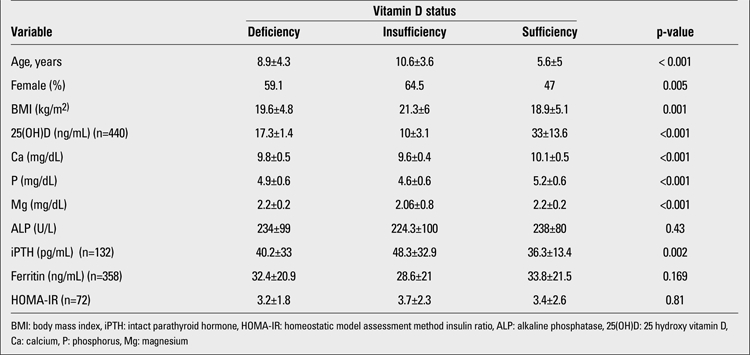
The anthtopmetric and metabolic characteristics of the study population

**Table 2:**
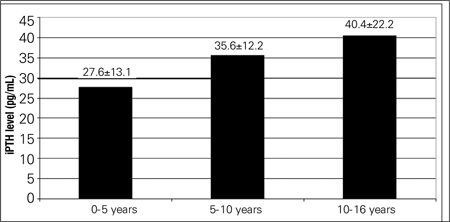
Pearson’s correlation coefficients of serum 25(OH)Dlevels with age, BMI, iPTH and ferritin levels, and HOMA-IR values
